# Supplementary material for: Bayesian association scan reveals loci associated with human lifespan and linked biomarkers
Source: Nat Commun. 2017 Jul 27;8:15842. doi: 10.1038/ncomms15842 (PMC5537485; doi:10.1038/ncomms15842)
Supplement: Supplementary Information [file ncomms15842-s1.pdf]

Type of file: PDF
Size of file: 0 KB
Title of file for HTML: Supplementary Information
Description: Supplementary Figures and Supplementary Tables

Type of file: pdf
File size:
Title of file for HTML: Peer Review File
Description:

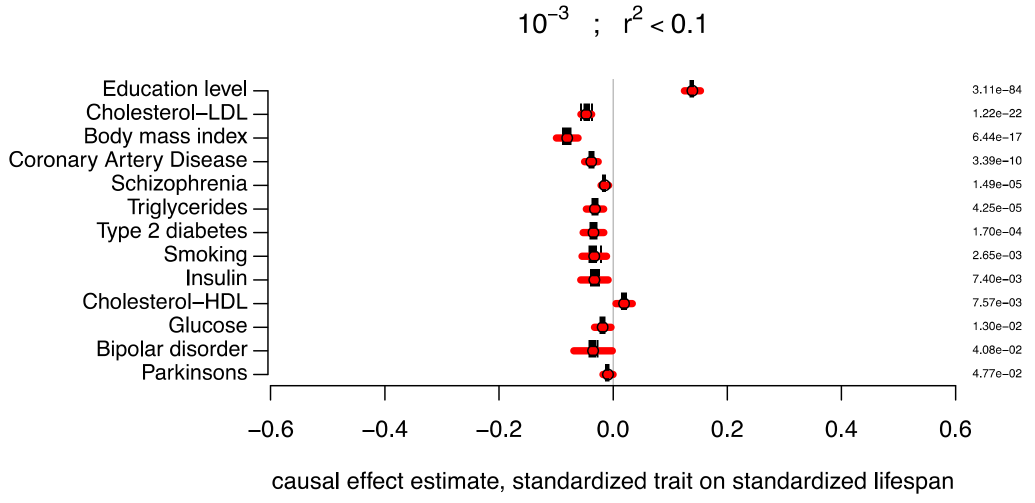
10^-3 ; r^2 < 0.1
Education level
Cholesterol–LDL
Body mass index
Coronary Artery Disease
Schizophrenia
Triglycerides
Type 2 diabetes
Smoking
Insulin
Cholesterol–HDL
Glucose
Bipolar disorder
Parkinsons
3.11e−84
1.22e−22
6.44e−17
3.39e−10
1.49e−05
4.25e−05
1.70e−04
2.65e−03
7.40e−03
7.57e−03
1.30e−02
4.08e−02
4.77e−02
−0.6
−0.4
−0.2
0.0
0.2
0.4
0.6
causal effect estimate, standardized trait on standardized lifespan

1

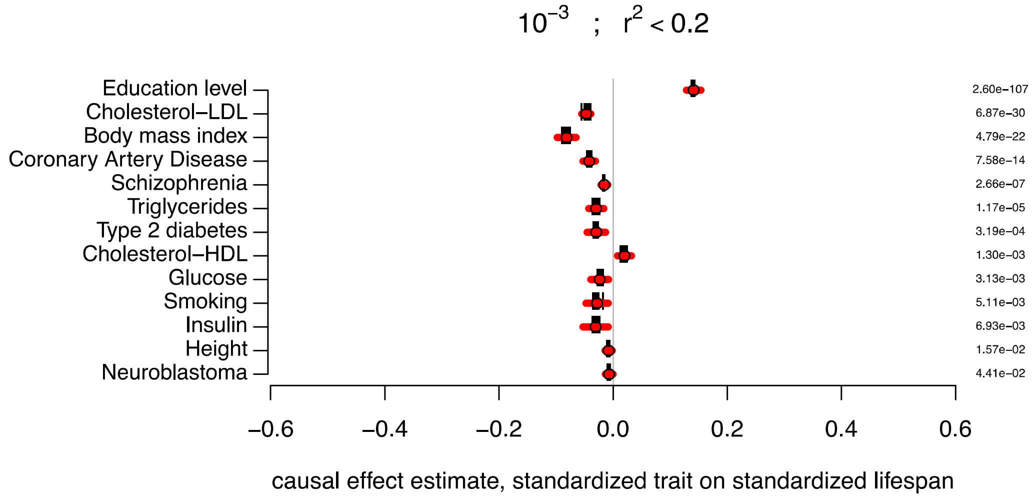
10^-3 ; r^2 < 0.2
Education level
Cholesterol–LDL
Body mass index
Coronary Artery Disease
Schizophrenia
Triglycerides
Type 2 diabetes
Cholesterol–HDL
Glucose
Smoking
Insulin
Height
Neuroblastoma
2.60e−107
6.87e−30
4.79e−22
7.58e−14
2.66e−07
1.17e−05
3.19e−04
1.30e−03
3.13e−03
5.11e−03
6.93e−03
1.57e−02
4.41e−02
−0.6
−0.4
−0.2
0.0
0.2
0.4
0.6
causal effect estimate, standardized trait on standardized lifespan

2

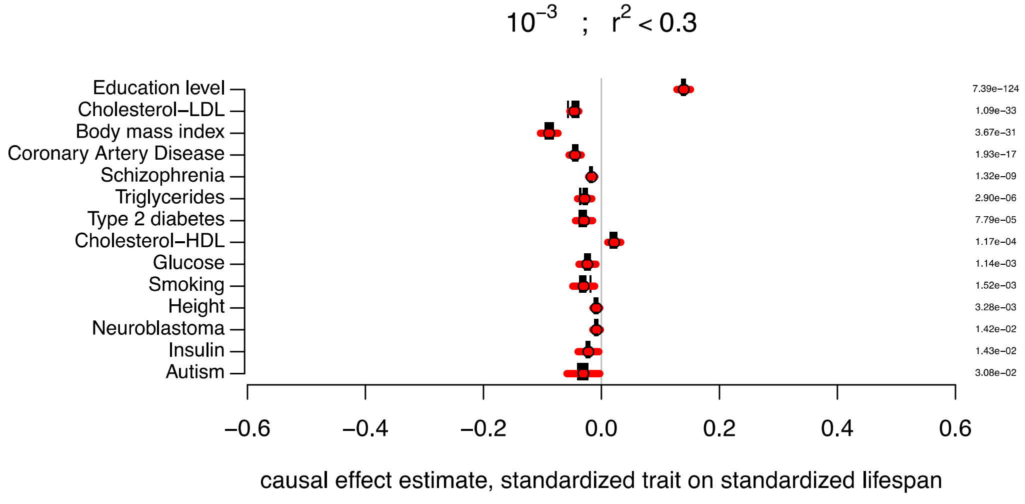
10^-3 ; r^2 < 0.3
Education level
Cholesterol–LDL
Body mass index
Coronary Artery Disease
Schizophrenia
Triglycerides
Type 2 diabetes
Cholesterol–HDL
Glucose
Smoking
Height
Neuroblastoma
Insulin
Autism
7.39e−124
1.09e−33
3.67e−31
1.93e−17
1.32e−09
2.90e−06
7.79e−05
1.17e−04
1.14e−03
1.52e−03
3.28e−03
1.42e−02
1.43e−02
3.08e−02
−0.6
−0.4
−0.2
0.0
0.2
0.4
0.6
causal effect estimate, standardized trait on standardized lifespan

3

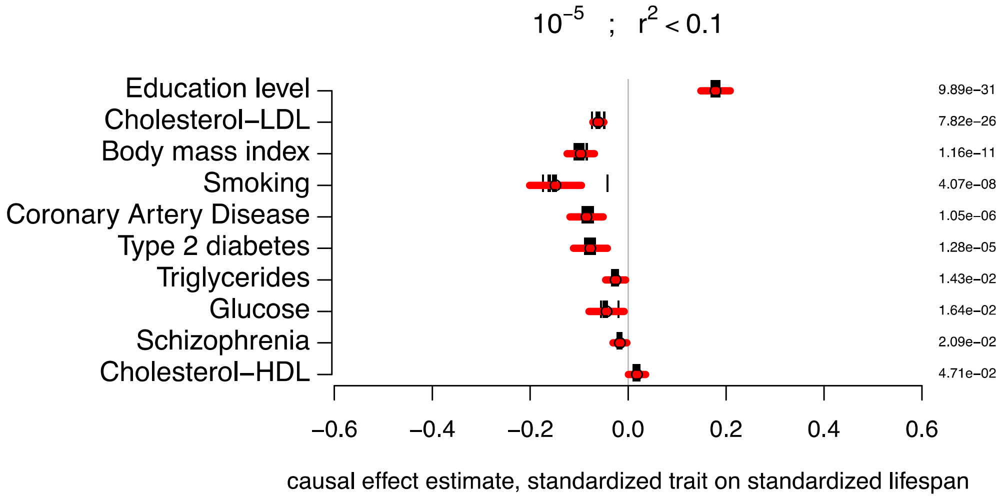

10^{-5} ; $r^2 < 0.1$
Education level
Cholesterol–LDL
Body mass index
Smoking
Coronary Artery Disease
Type 2 diabetes
Triglycerides
Glucose
Schizophrenia
Cholesterol–HDL
9.89e–31
7.82e–26
1.16e–11
4.07e–08
1.05e–06
1.28e–05
1.43e–02
1.64e–02
2.09e–02
4.71e–02
–0.6 –0.4 –0.2 0.0 0.2 0.4 0.6
causal effect estimate, standardized trait on standardized lifespan

4

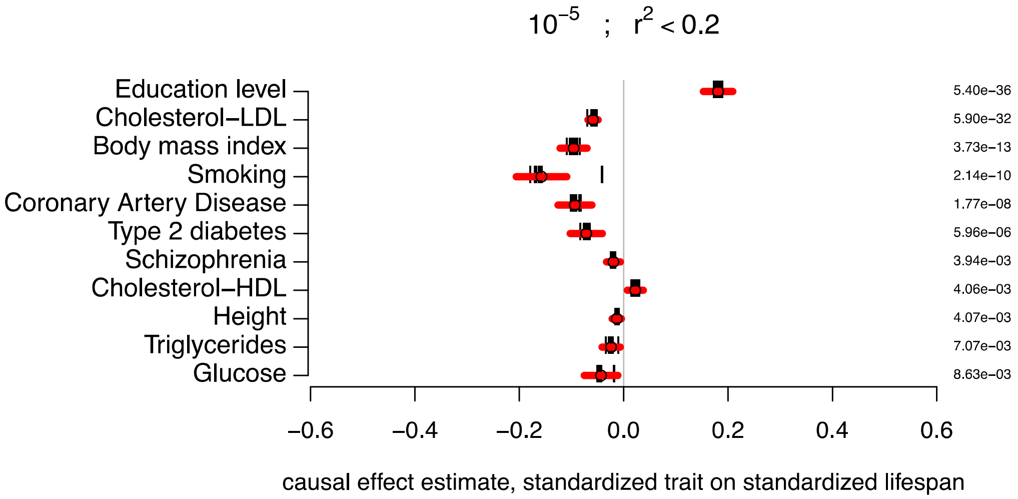

10^{-5} ; $r^2 < 0.2$
Education level
Cholesterol–LDL
Body mass index
Smoking
Coronary Artery Disease
Type 2 diabetes
Schizophrenia
Cholesterol–HDL
Height
Triglycerides
Glucose
5.40e–36
5.90e–32
3.73e–13
2.14e–10
1.77e–08
5.96e–06
3.94e–03
4.06e–03
4.07e–03
7.07e–03
8.63e–03
–0.6 –0.4 –0.2 0.0 0.2 0.4 0.6
causal effect estimate, standardized trait on standardized lifespan

5

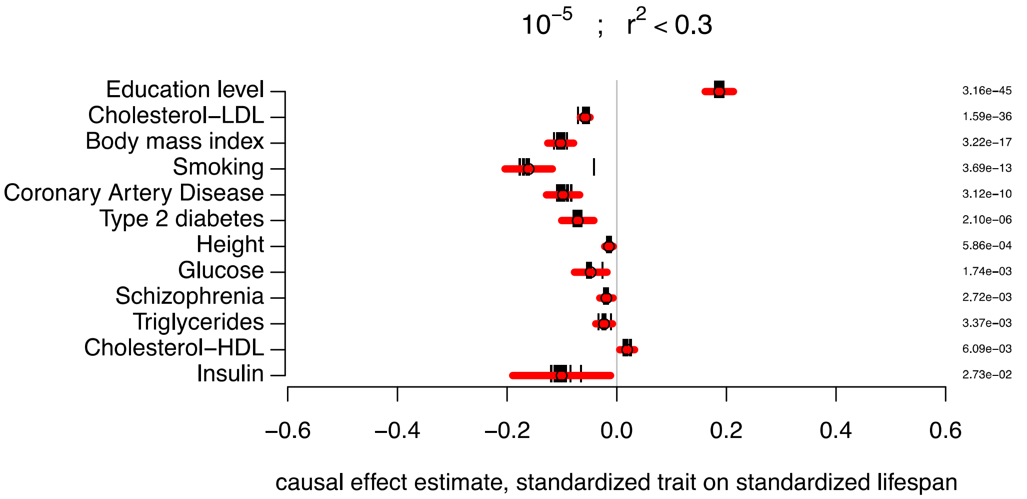

10^{-5} ; $r^2 < 0.3$
Education level
Cholesterol–LDL
Body mass index
Smoking
Coronary Artery Disease
Type 2 diabetes
Height
Glucose
Schizophrenia
Triglycerides
Cholesterol–HDL
Insulin
3.16e–45
1.59e–36
3.22e–17
3.69e–13
3.12e–10
2.10e–06
5.86e–04
1.74e–03
2.72e–03
3.37e–03
6.09e–03
2.73e–02
–0.6 –0.4 –0.2 0.0 0.2 0.4 0.6
causal effect estimate, standardized trait on standardized lifespan

6

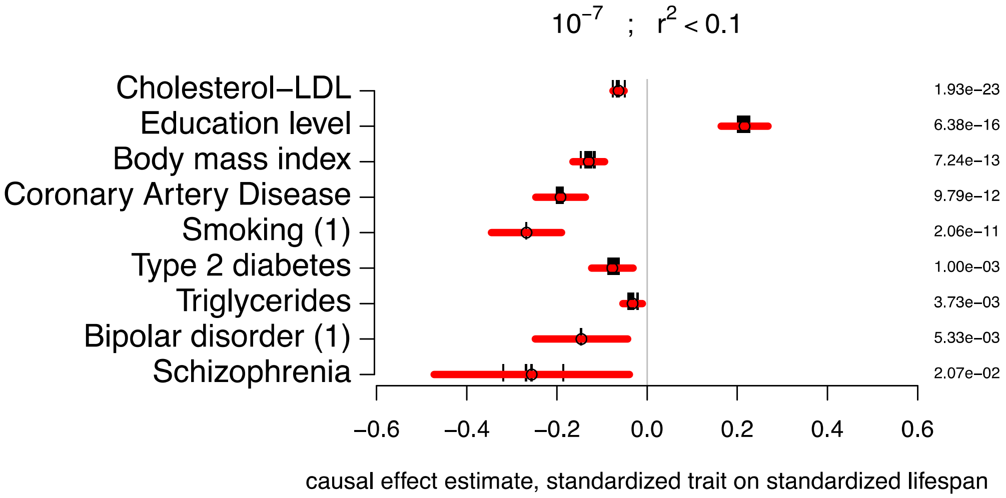
10^-7 ; r^2 < 0.1
Cholesterol−LDL
Education level
Body mass index
Coronary Artery Disease
Smoking (1)
Type 2 diabetes
Triglycerides
Bipolar disorder (1)
Schizophrenia
1.93e−23
6.38e−16
7.24e−13
9.79e−12
2.06e−11
1.00e−03
3.73e−03
5.33e−03
2.07e−02
−0.6 −0.4 −0.2 0.0 0.2 0.4 0.6
causal effect estimate, standardized trait on standardized lifespan

7

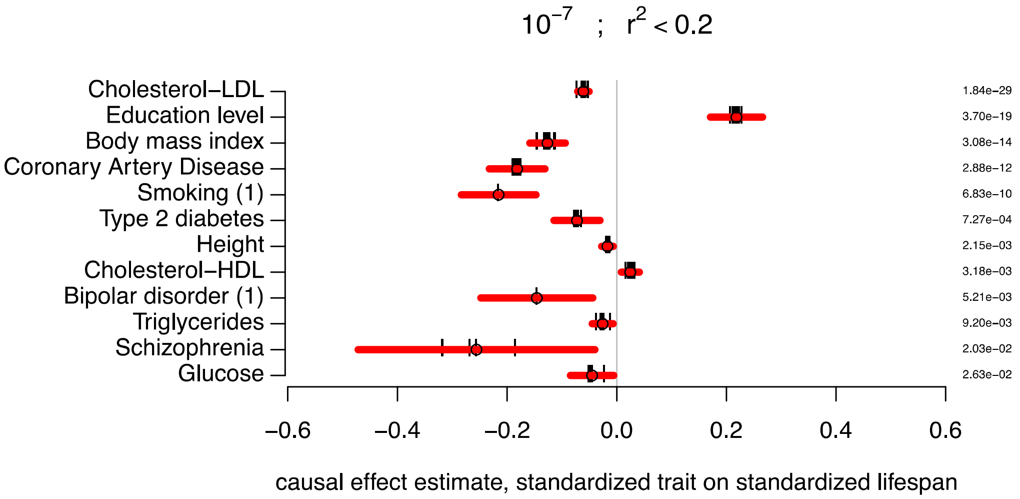
10^-7 ; r^2 < 0.2
Cholesterol−LDL
Education level
Body mass index
Coronary Artery Disease
Smoking (1)
Type 2 diabetes
Height
Cholesterol−HDL
Bipolar disorder (1)
Triglycerides
Schizophrenia
Glucose
1.84e−29
3.70e−19
3.08e−14
2.88e−12
6.83e−10
7.27e−04
2.15e−03
3.18e−03
5.21e−03
9.20e−03
2.03e−02
2.63e−02
−0.6 −0.4 −0.2 0.0 0.2 0.4 0.6
causal effect estimate, standardized trait on standardized lifespan

8

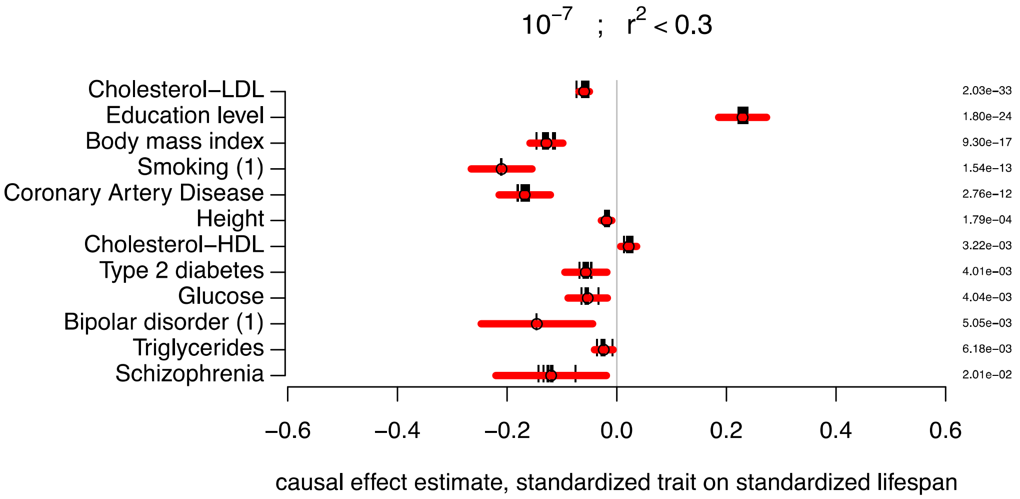
10^-7 ; r^2 < 0.3
Cholesterol−LDL
Education level
Body mass index
Smoking (1)
Coronary Artery Disease
Height
Cholesterol−HDL
Type 2 diabetes
Glucose
Bipolar disorder (1)
Triglycerides
Schizophrenia
2.03e−33
1.80e−24
9.30e−17
1.54e−13
2.76e−12
1.79e−04
3.22e−03
4.01e−03
4.04e−03
5.05e−03
6.18e−03
2.01e−02
−0.6 −0.4 −0.2 0.0 0.2 0.4 0.6
causal effect estimate, standardized trait on standardized lifespan

9

Supplementary Figure 1. The causal effect estimates vary as we change the set of instruments used in the multivariate MR regression. As described in the Methods section, the instruments are first filtered on a p-value (10^{-3},10^{-5}, or 10^{-7}, applied to any of the non-lifespan traits) and then pruned with an LD threshold for identifying independent instruments ($r^2 < 0.1$, $r^2 < 0.2$, $r^2 < 0.3$). The nine plots above demonstrate that, while the set of traits selected by AIC changes, the direction and approximate magnitude of the effects is independent of the filtering and pruning parameters just described.

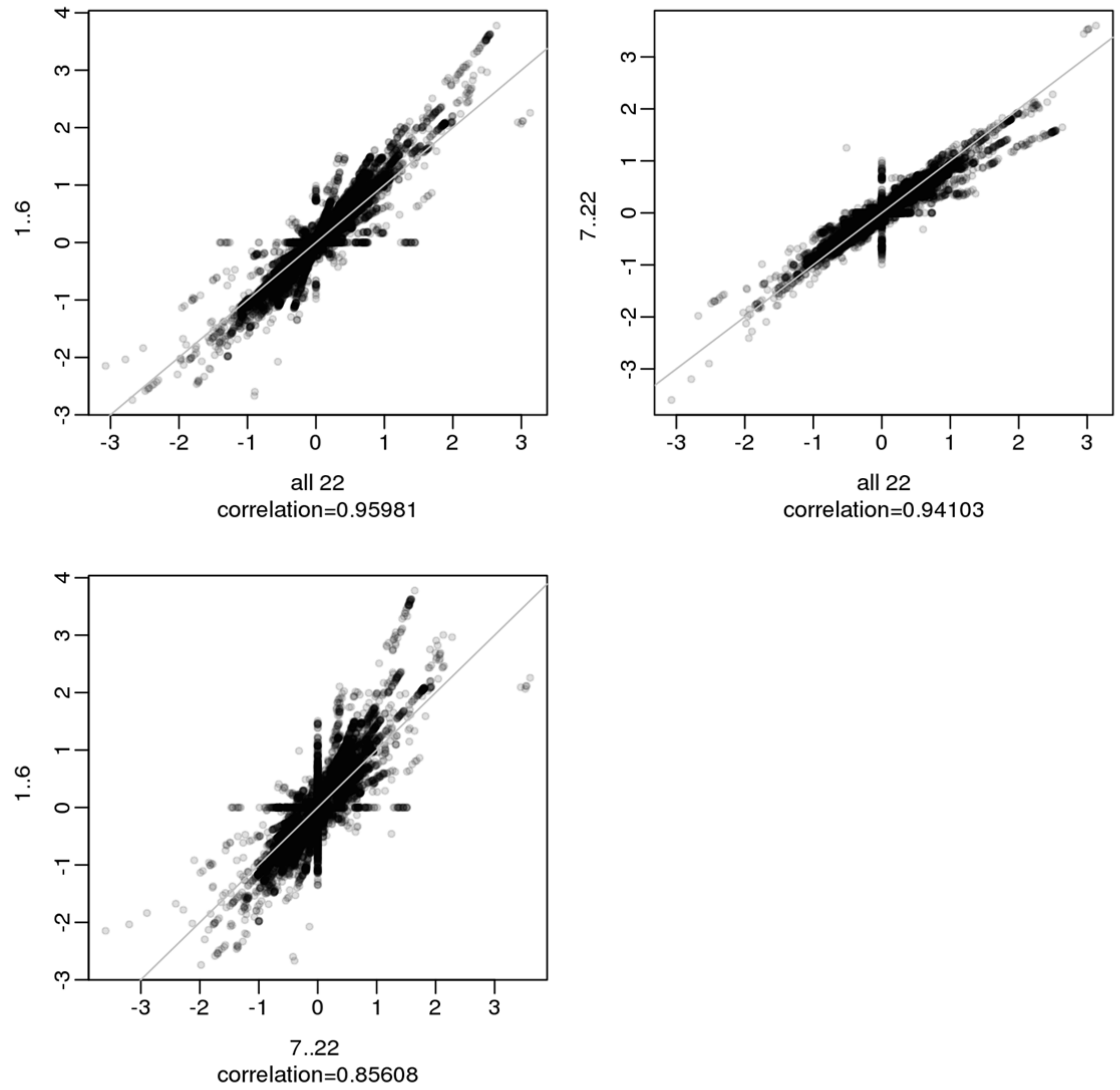

Supplementary Figure 2. We compute the priors for all 22 chromosomes, based on causal effect estimates from 21 chromosomes under the leave-one-chromosome-out scheme. We also recomputed priors across all 22 chromosomes using only the first six chromosomes, where only six chromosomes (or five chromosomes, due to the leave-one-chromosome-out scheme) were used to estimate causal effects. Further, we recomputed priors where only the last 16 chromosomes were available for causal effect estimation. This gives us three sets of priors, which are highly correlated with each other as demonstrated in this plots. (subsection “Possible causal effects of GWAS traits on parental lifespan”).

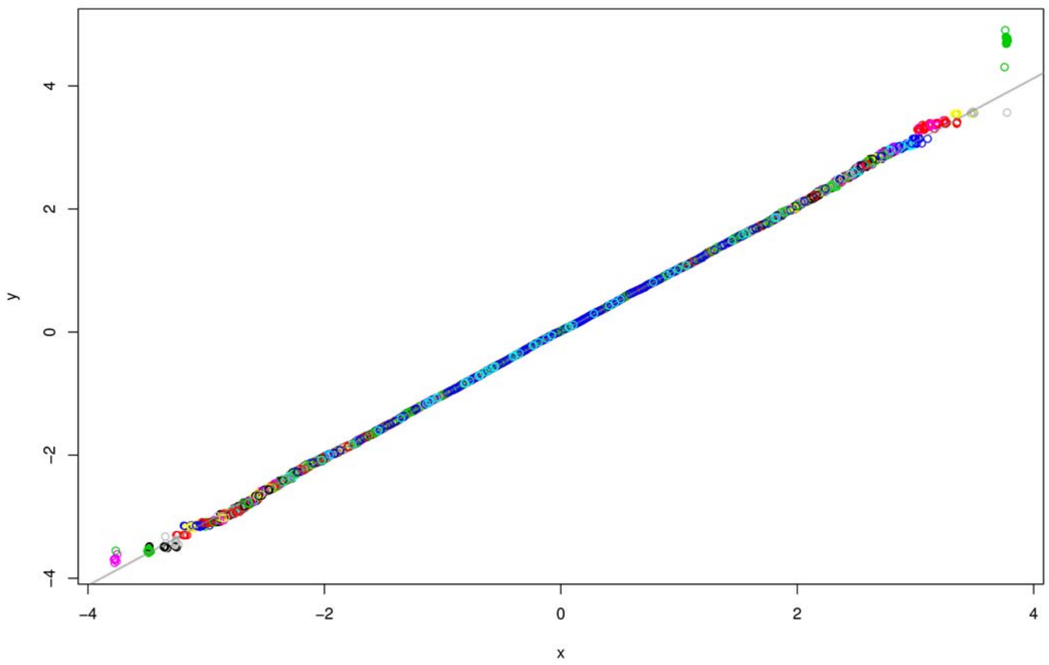

Supplementary Figure 3. 22 qq-plots of the residuals of the linear regressions performed for the causal effect estimates. 22 regressions were performed, where each of the 22 chromosomes was left out in turn, hence 22 qq-plots each with a different colour. The residuals show the expected standard normal distribution (scale of z-statistic) except for one SNP near APOE. The lack of outliers indicate little evidence for instrument pleiotropy in the MR analysis.

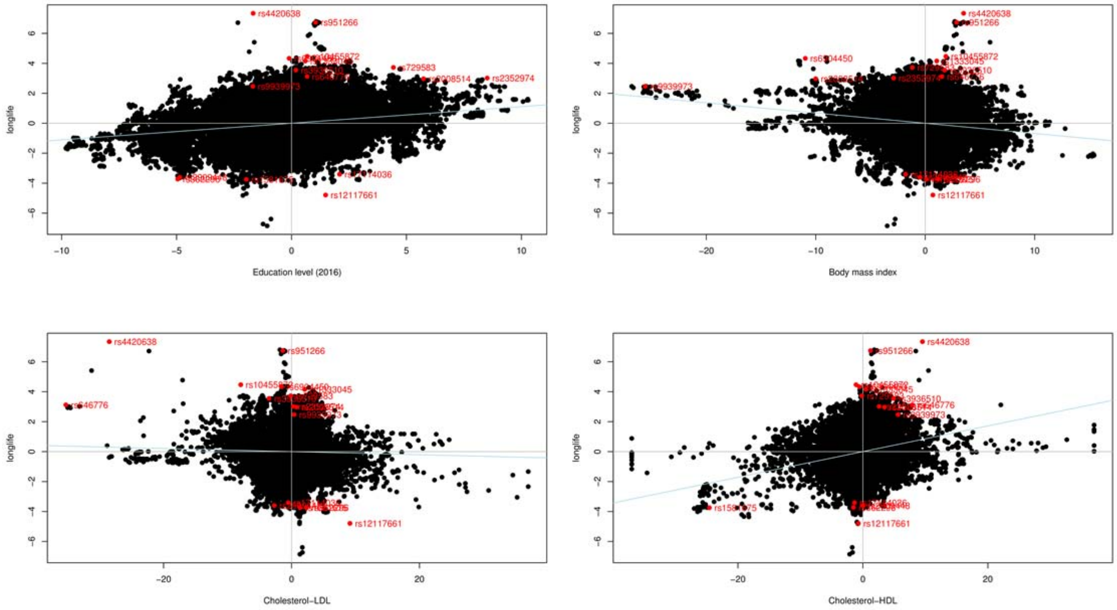

Supplementary Figure 4. A scatter plot for each of four traits (Education level, BMI, Cholesterol-LDL, Cholesterol-HDL) showing the trait's z-statistic (x-axis) against the z-statistic for parental lifespan (y-axis) for the 6,443 SNPs used as instruments.

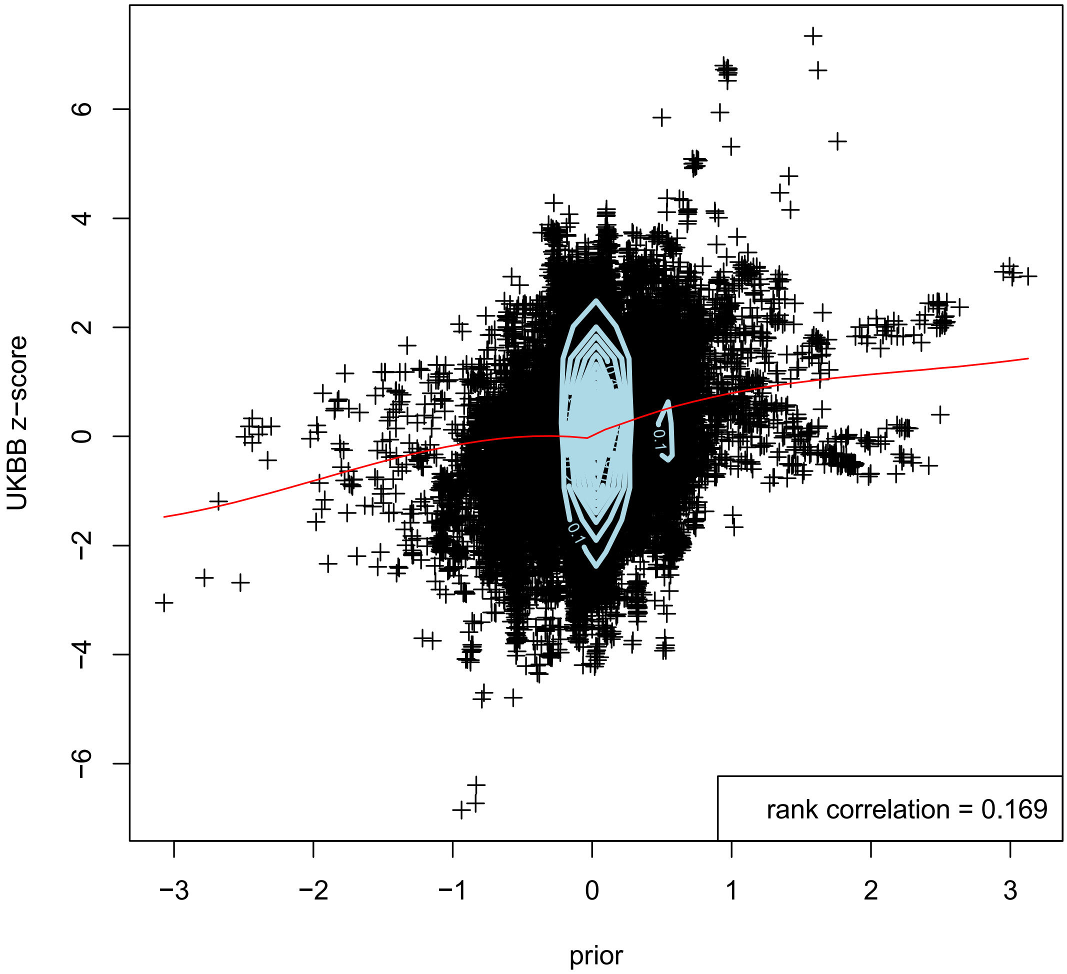

Supplementary Figure 5. Scatter plot of the computed prior (Methods section, scaled to z-statistic-scale) against the z-statistic observed in the UKBB parental lifespan. The priors are constructed such that they are formally independent of the z-statistics, due to the leave-one-chromosome-out scheme. The positive correlation demonstrated here confirms that the priors are informative.

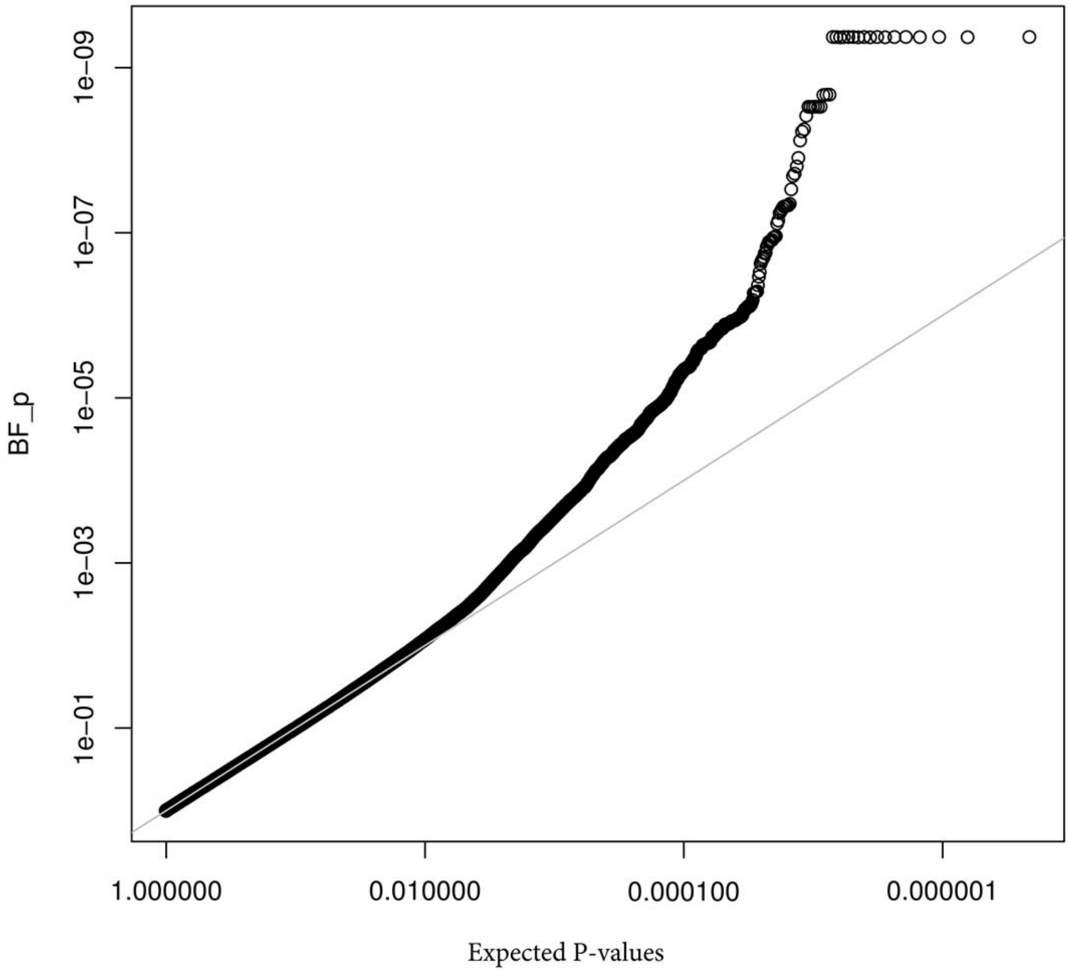

Supplementary Figure 6. QQ-plot of the P-values computed by comparing the observed Bayes Factors to a large collection of "null" Bayes Factors computed by simulation.

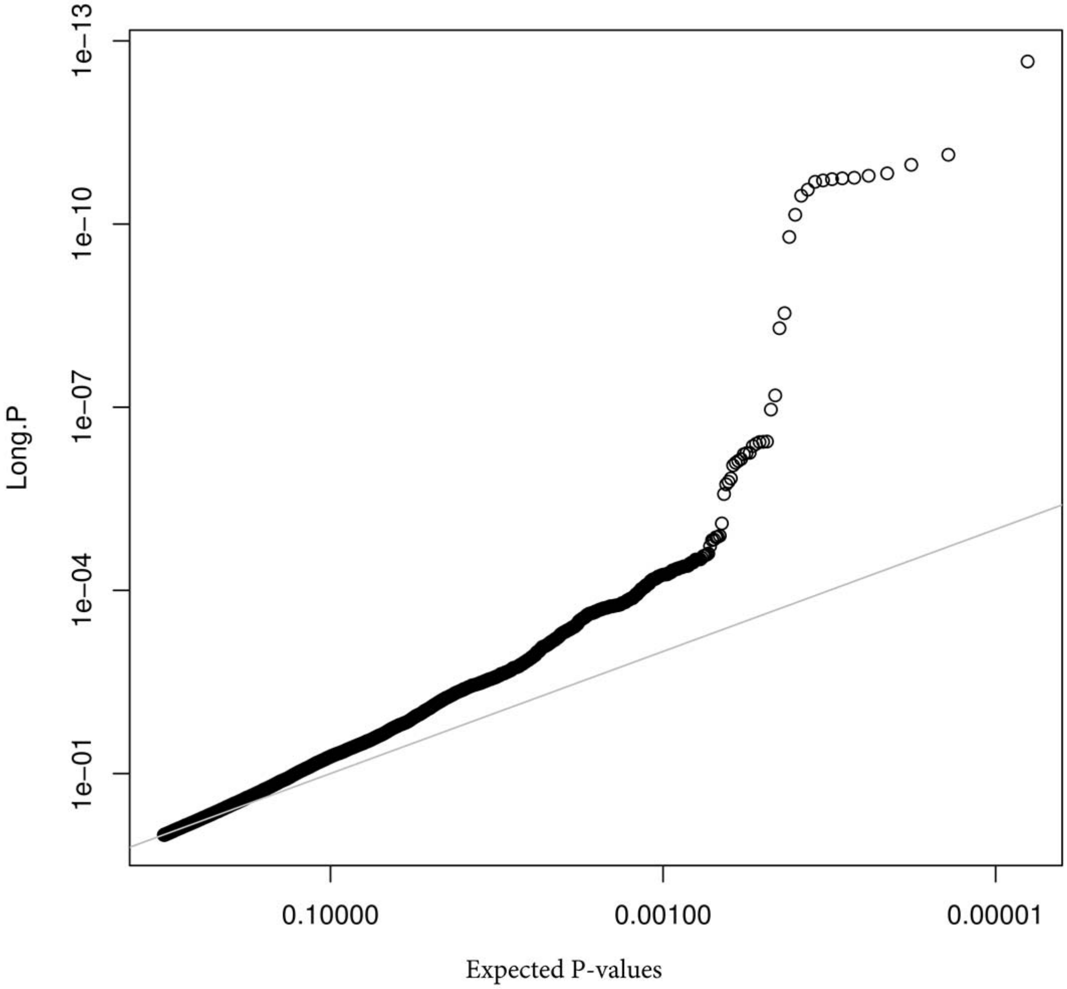

Supplementary Figure 7. QQ-plot of the UK Biobank P-values restricted to the 77,963 SNPs that had a non-zero prior.

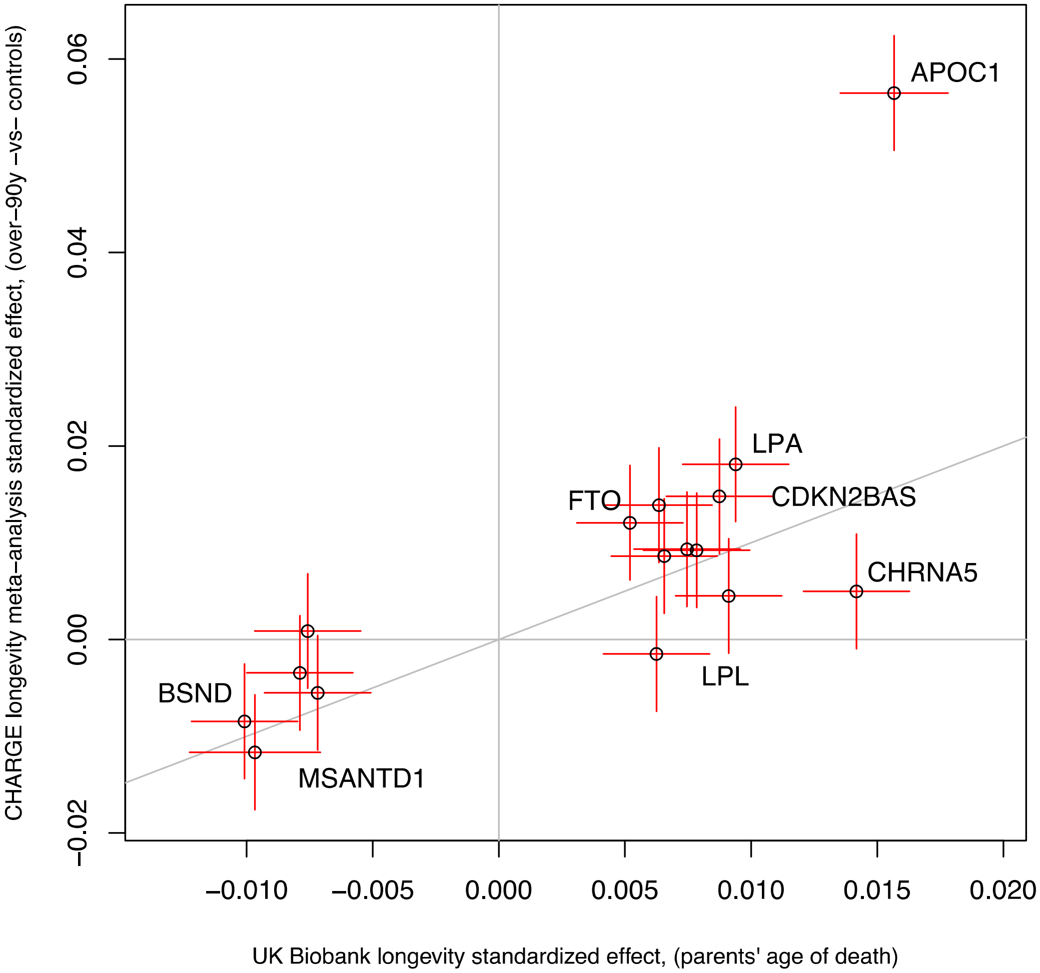

Supplementary Figure 8. Scatter plot of the GWAS of the "continuous" lifespan trait (UKBB parental lifespan, x-axis) versus the binary longevity analysis of an independent study (CHARGE case-control analysis, y-axis).

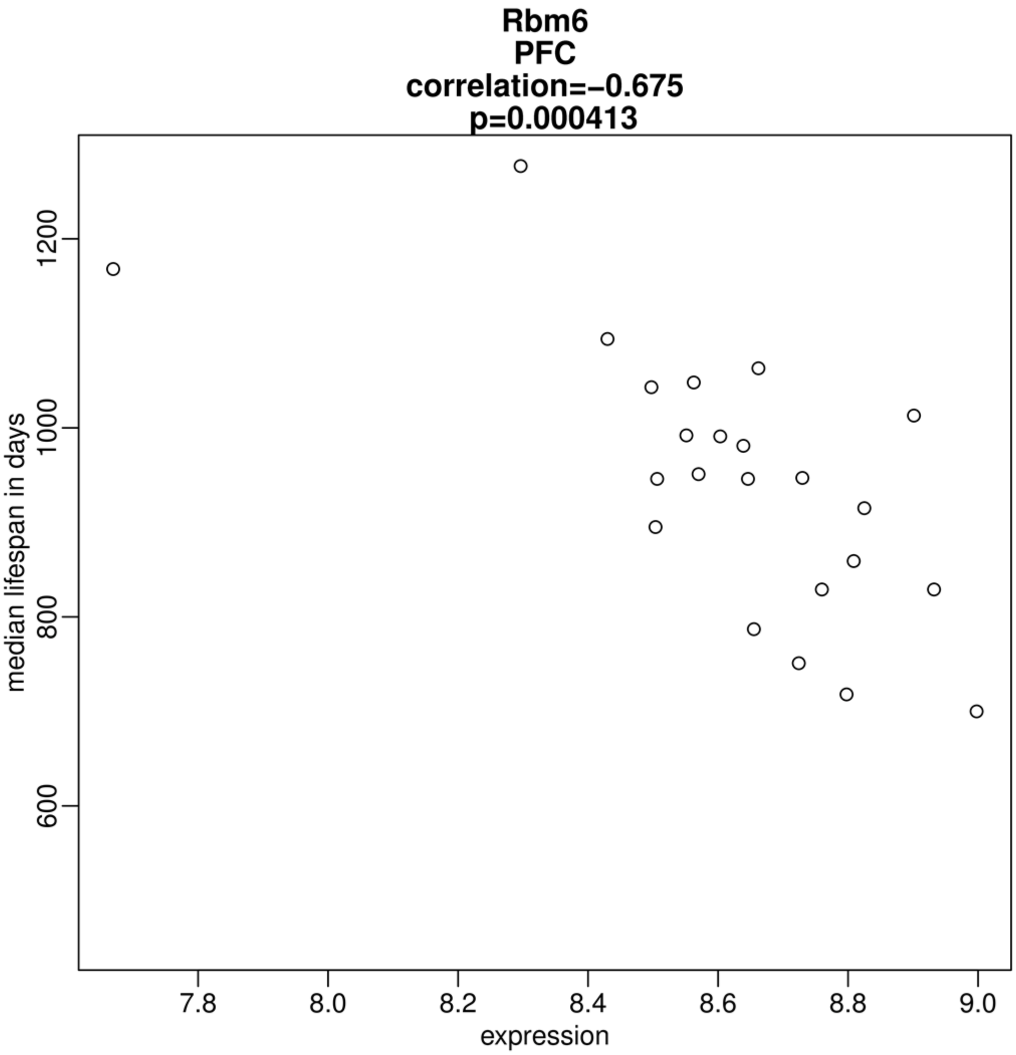

Supplementary Figure 9. Scatter plot of expression level of RBM6 (prefrontal cortex, taken at 72 days of age) in 35 strains of LXS mouse lines, against median lifespan of that strain.

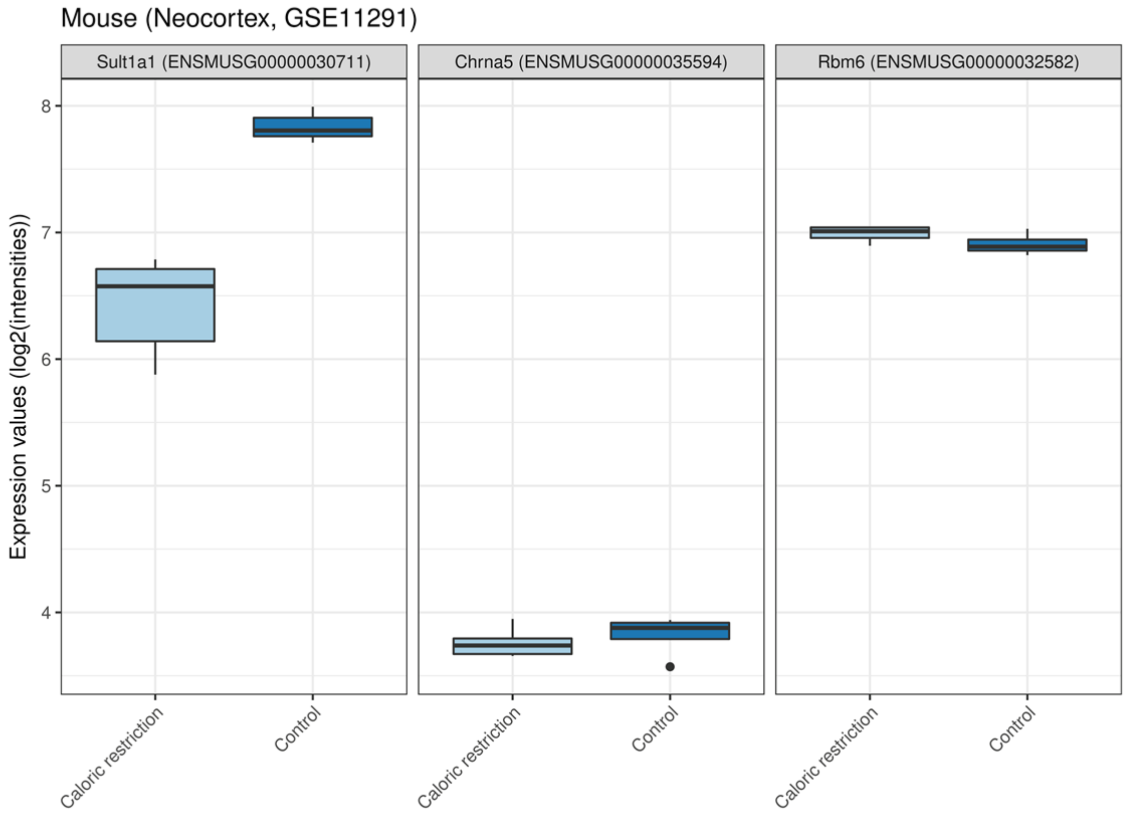

Supplementary Figure 10. Expression levels for three genes of interest (*SULT1A1, CHRNA5*, and *RBM6*) in mice, comparing mice under caloric restriction (left of each subplot) to controls (right of each subplot).

Trait	effect estimate using instruments from chr 1-6	effect estimate using instruments from chr 7-22	effect estimate using instruments from chr 1-22	effect estimate using instruments from chr 1-22 with non-zero intercept
**Body mass index**	**-0.139093151**	**-0.067460144**	**-0.095771617**	**-0.09565591**
**Education level (2016)**	**0.198353558**	**0.162571806**	**0.180982724**	**0.18112609**
**Glucose**	**-0.072218066**	**NA**	**-0.04331209**	**-0.04321186**
**Height**	**-0.019793819**	**NA**	**-0.013064918**	**-0.01304632**
**Type 2 diabetes**	**-0.115314614**	**-0.057136871**	**-0.07159749**	**-0.07150896**
**Coronary Artery Disease**	**-0.105779408**	**-0.091903707**	**-0.093368218**	**-0.0935108**
**Cholesterol-HDL**	**NA**	**0.024641134**	**0.022341532**	**0.02240102**
**Cholesterol-LDL**	**-0.051466106**	**-0.066286425**	**-0.058746978**	**-0.05875607**
**Triglycerides**	**-0.046124696**	**NA**	**-0.023998349**	**-0.02405075**
**Schizophrenia**	**-0.027933396**	**NA**	**-0.019571358**	**-0.01961813**
**Smoking (cigarette per day)**	**NA**	**-0.163254383**	**-0.157455484**	**-0.15744647**

Supplementary Table 2. Comparing the causal effect estimates computed by the linear regression under four different scenarios. The first column uses only instruments from the first six chromosomes. The second column is based on the remaining 16 chromosomes. The third column uses all 22 chromosomes. For the fourth column, an intercept term was included in the regression; every other regression in this paper did not include an intercept term. See also Supplementary Figure 2.

SNP	Z-stat UKB parental lifespan	P UKB parental lifespan	P iGWAS NECS (proxy SNP where necessary)	P iGWAS NECS	P iGWAS 90plus	p.Walt.CR G	p.CRGUS	p.CRG90	p-value (compared to Fisher-statistic on null SNPs	FDR (Benjamini – Hochberg)
rs12117661	4.79201	1.65E-06	1	NA	0.000236	0.288155	0.6233	0.029895	0.00271	0.005178
rs17114036	3.407074	0.000657	0.667	NA	1	0.451294	0.2606	0.050543	0.324763	0.346414
rs646776	3.119192	0.001813	0.000566	0.000566	0.00058	0.143479	0.1773	0.332574	0.000662	0.002119
rs2909448	3.60213	0.000316	1	1	1	0.230607	0.8667	0.761726	0.252491	0.288561
rs2352974	3.01798	0.002545	1	NA	1	0.006759	0.1565	0.038553	0.011911	0.017325
rs362296	3.729171	0.000192	1	NA	1	0.079087	0.7877	0.018449	0.092898	0.123864
rs3936510	3.546957	0.00039	0.0244	NA	0.0403	0.103341	0.4182	0.137229	0.002913	0.005178
rs6904450	4.33114	1.48E-05	0.708	NA	0.0521	0.044256	0.1417	0.992323	0.005565	0.008904
rs10455872	4.465442	7.99E-06	0.00563	NA	2.72E-06	0.000921	0.2002	0.002085	6.98E-05	0.000372
rs1581675	3.74875	0.000178	0.517	NA	1	0.288668	0.7232	0.322542	0.167265	0.205864
rs1333045	4.15489	3.25E-05	1.69E-06	NA	5.79E-05	0.001069	0.07957	0.041273	3.17E-05	0.000254
rs951266	6.739144	1.59E-11	0.000854	NA	1	0.025345	0.3521	0.648223	0.002342	0.005178
rs729583	3.72611	0.000194	1	NA	1	0.337017	0.2431	0.221467	0.361454	0.361454
rs2008514	2.96983	0.00298	0.00266	NA	0.131	0.106804	0.9348	0.787709	0.002528	0.005178
rs9939973	2.46999	0.013512	0.00149	NA	0.000139	0.015675	0.1473	0.098983	0.000222	0.000888
rs4420638	7.339788	2.14E-13	3.68E-13	NA	5.73E-24	2.61E-13	2.13E-05	4.09E-21	2.12E-06	3.38E-05

Supplementary Table 3. Replication by looking up our 16 hits in a meta-analysis of other longevity-related studies. Those other studies have some sample overlap with each other, but not with the UK Biobank, hence they can be used as a replication sample as long as this overlap is accounted for correctly. Three of the studies are combined by the method of Lin et al to combine effect estimates in the presence of sample overlap, and then the resulting p-value is combined (via Fisher's method) with p-values from each of the two Fortney et al datasets. The resulting "Fisher statistic" is then compared against the distribution of this same statistic computed across all (null) SNPs to allow us to identify significant Fisher statistics and assign a p-value (second to last column) to each of the 16 observed Fisher statistics. After adjusting those "permutation" p-values by the Benjamini-Hochberg method (final column), 11 of the 16 are significant at 5% FDR.

Study	Cohort	rs	chr	pos	gene	effect allele	HR in the study indicated in column A	P-value in the study indicated in column A	Z-statistic in our UK Biobank study
Walter et al		rs4936894	11	123522703	VWA5A	A	1.11	3.38E-07	0.87297
Walter et al		rs1425609	3	164164689	OTOL1	A	0.92	1.46E-06	0.821068
Walter et al		rs766903	12	49990101	BIN2	A	0.9	1.61E-06	0.528719
Walter et al		rs12042640	1	63139384	ATG4C	T	1.09	1.71E-06	1.30556
Walter et al		rs17149227	7	75073485	HIP1	T	0.79	3.56E-06	-0.317475
Walter et al		rs3128591	9	136741940	COL5A1	A	0.92	3.64E-06	-0.904607
Walter et al		rs11582903	1	87618642	LMO4	A	1.12	3.94E-06	-0.853689
Walter et al		rs4850695	2	196861504	HECW2	A	1.09	4.62E-06	NA
Walter et al		rs10259086	7	103680248	ORC5L	T	1.08	5.16E-06	NA
Walter et al		rs2769255	1	41017941	KCNQ4	T	1.08	5.17E-06	0.113546
Walter et al		rs17291546	6	2660681	LOC340156	A	0.82	7.65E-06	0.631934
Walter et al		rs12606100	18	69102967	NETO1	T	1.11	8.72E-06	-1.35348
Walter et al		rs1274214	11	122979741	GRAMD1B	T	0.93	8.87E-06	-0.502468
Walter et al		rs10811679	9	2224701	SMARCA2	T	1.08	9.53E-06	0.256602
Fortney et al	NECS	rs2075650	19	50087458	TOMM40/APOE	A	0.48	3.68E-13	5.40907
Fortney et al	NECS	rs4977756	9	22058651	CDKN2B/ANRIL	G	0.73	2.46E-08	2.78889
Fortney et al	NECS	rs3763305	6	32477465	HLA	A	0.53	9.78E-08	-0.643203
Fortney et al	NECS	rs3184504	12	110368990	SH2B3/ATXN2	G	0.79	7.88E-07	3.63911
Fortney et al	90PLUS	rs4420638	19	50114785	TOMM40/APOE	A	0.64	5.73E-24	7.33979
Fortney et al	90PLUS	rs514659	9	135132023	ABO	A	0.89	2.51E-07	0.847333
Fortney et al	90PLUS	rs10737670	1	194624368	KCNT2	G	0.9	1.29E-06	-2.19798
Fortney et al	90PLUS	rs12194148	6	32552175	HLA	G	0.91	1.57E-06	
Fortney et al	90PLUS	rs174555	11	61336335	FADS1	C	0.91	1.56E-06	1.93018
Fortney et al	90PLUS	rs10455872	6	160930107	LPA	A	0.84	2.72E-06	4.46544

Supplementary Table 4. Lookup of candidates suggested from other studies (14 from Walter et al, and 9 SNPs from Fortney et al) to check if they are significant in the UK Biobank parental lifespan study (final column).

Locus	TISSUE	GENE	BETA (causal effect estimate)	SE(BETA)	P-value (causal effect estimate)	N snps
16p11.2	BRAIN	SULT1A1	-0.014140143	0.00206141	6.91E-12	16
15q25.1	BRAIN	CHRNA5	-0.012886558	0.00293671	1.14E-05	20
3p21.31	BRAIN	RBM6	-0.012062694	0.00361517	0.000847841	8

Supplementary Table 5. Causal effect estimates of gene expression on lifespan in mice. In each cases, a number of SNPs (the last column 'N snps') were used simultaneously to create a single causal effect estimate.

gene symbol	Spearman correlation between expressionvalue and lifespan	Spearman correlation P-value	mixed model P-value
RBM6	-0.674740567	0.000413053	0.000115421
CHRNA5	0.171527455	0.433880386	0.208001833
SULT1A1	-0.155709362	0.478040638	0.841265582

Supplementary Table 6. Simple correlation estimates between expression level and lifespan in mice. Third column uses a mixed model to correct for population structure.

SNP ID	Other Allele	Effect Allele	Z statistic	chromosome	position	prior	SE prior	Log(BF)	BF_p	P
rs4420638	A	G	-7.340	19	45422946	1.586	0.422	12.803	4.25E-10	2.14E-13
rs12117661	C	G	4.792	1	55487346	-0.567	0.362	3.530	5.10E-07	1.65E-06
rs10519203	G	A	-6.739	15	78878541	0.978	0.888	13.142	4.25E-10	1.59E-11

Supplementary Table 7. Loci surviving 5% FDR based on their UK Biobank P-values if the analysis were restricted to the 77,963 SNPs SNPs with non-zero prior.

Setting	Name	P-value (chi2)	P-value (emp)
max	REACTOME_LIPOPROTEIN_METABOLISM	9.81E-06	2.80E-06
max	REACTOME_LIPID_DIGESTION_MOBILIZATION_AND_TRANSPORT	1.34E-04	2.18E-05
max	REACTOME_CHYLOMICRON_MEDIATED_LIPID_TRANSPORT	9.59E-05	4.66E-05
sum	REACTOME_LIPOPROTEIN_METABOLISM	4.03E-06	3.10E-06
sum	REACTOME_CHYLOMICRON_MEDIATED_LIPID_TRANSPORT	5.06E-06	3.60E-06
sum	REACTOME_LIPID_DIGESTION_MOBILIZATION_AND_TRANSPORT	2.87E-05	1.47E-05

Supplementary Table 8. Pathways with PASCAL enrichment P-value (based on lifespan BF permutation P-values) suriving 5% FDR.
